# Functional Outcome in Obese Patients Undergoing Image-Based Cruciate Retaining Robotic-Assisted Total Knee Arthroplasty Using the Subvastus Approach: A Short-Term Study

**DOI:** 10.7759/cureus.68430

**Published:** 2024-09-02

**Authors:** Sujoy K Bhattacharjee, Arghya Kundu Choudhury, Swapnil Priyadarshi, Avijeet Prasad, Akhil Ahlawat

**Affiliations:** 1 Robotics and Joint Replacement, Sarvodaya Hospital and Research Centre, Faridabad, IND

**Keywords:** mini-subvastus approach, obese patient, total knee arthroplasty technique, robotic-arm assisted surgery, knee osteoarthritis/ koa

## Abstract

Introduction

Knee osteoarthritis (OA) is a prevalent degenerative joint disease that significantly affects quality of life, especially among obese and morbidly obese individuals. Total knee arthroplasty (TKA) is an effective treatment for end-stage OA, but it presents unique challenges in obese patients. The mini-subvastus approach (SA) and robotic-assisted TKA (RA-TKA) are emerging techniques that may address these challenges. This study evaluates the clinical and radiological outcomes of cruciate-retaining (CR) RA-TKA using the mini-subvastus approach in obese and morbidly obese patients.

Methods

This study included 114 obese patients (215 knees) with primary OA who underwent CR RA-TKA (Maxx Meril CR knee, USA) using the Cuvis Joint® robotic system. Patients had a BMI of ≥30 kg/m² (n=101) and morbid obesity with a BMI ≥40 kg/m² (n=13). Preoperative planning involved CT scans and the J-planner for optimal implant size and positioning. Surgery was performed without tourniquets, and patients were mobilized postoperatively. Clinical outcomes were assessed using visual analog scale (VAS) scores and the American Knee Society Score (AKSS) at three and six months.

Results

The study included 85 females and 29 males with an average age of 61.8 years. Satisfactory knee exposure was achieved in all cases using SA, with no major complications such as wound infections, deep vein thrombosis, or component misalignment. Intraoperative challenges were managed effectively, including two cases of medial collateral ligament avulsion and one partial patellar tendon avulsion. Postoperative VAS scores showed significant pain reduction from pre-op (6.54) to post-op day 3 (1.3). AKSS improved from a pre-op average of 33.9 to 70.7 at three months and 80.9 at six months. Most patients (80%) achieved exceptional range of motion (ROM) ≥120° at six months.

Discussion

The mini-subvastus approach in RA-TKA offers several advantages, including reduced postoperative pain, faster recovery, and improved quadriceps strength, even in obese patients. The use of robotic assistance ensures accurate component positioning and alignment, mitigating the challenges typically associated with obese patients undergoing TKA.

Conclusion

The study demonstrates the feasibility and effectiveness of CR RA-TKA using the mini-subvastus approach in obese and morbidly obese patients. This technique provides adequate exposure, reduces pain, and promotes early mobilization and recovery with satisfactory clinical and radiological outcomes. The findings support the potential for wider adoption of this approach in managing knee OA in obese populations, though further studies with longer follow-up are warranted.

## Introduction

Knee osteoarthritis (OA) is a degenerative joint disease that affects millions of people worldwide and causes pain, stiffness, and reduced function [1]. Obesity is one of the major risk factors for knee OA, as it increases the mechanical load and inflammatory mediators in the joint. The excess body weight in obese individuals places increased stress on the knee joints, particularly the weight-bearing surfaces like the cartilage and subchondral bone. Furthermore, obesity is associated with systemic inflammation due to the production of pro-inflammatory cytokines and adipokines from adipose tissue. These inflammatory mediators can exacerbate joint inflammation, contributing to pain and the further breakdown of cartilage. According to the World Health Organization (WHO), obesity is defined as a body mass index (BMI) ≥ 30 kg/m^2^ and morbid obesity as a BMI ≥ 40 kg/m^2^ [2]. The prevalence of knee OA is higher in obese and morbidly obese populations, especially in women and Asians [3].

Total knee arthroplasty (TKA) is an effective treatment for end-stage knee OA, providing pain relief and restoring function [4]. However, performing TKA in obese and severely obese patients presents challenges, including limited exposure, increased blood loss, longer operative time, higher complication rates, and lower patient satisfaction [5].

In orthopaedic surgery, there has been a growing emphasis on minimally invasive and muscle-sparing techniques. Even though no concrete explanations have been provided, it is thought that obese patients undergoing TKR should not be operated on using the subvastus technique. Although issues with patella eversion, intraoperative visibility, and component alignment in the subvastus technique have been brought up, encouraging findings have recently been published that point to better early recovery with comparable complication rates using the mini-subvastus approach (SA). We hypothesized that obesity would not significantly affect the outcomes for patients undergoing TKA using the mini-subvastus approach [6].

Studies have shown that the mini-SA reduces postoperative pain, improves quadriceps strength, enables early mobilization, and shortens hospital stays compared to the conventional medial parapatellar approach as it preserves quadriceps muscle integrity [6,7]. Importantly, SA can be safely and effectively performed in obese and morbidly obese patients without compromising exposure or increasing the risk of complications [8].

Another possible solution is to use a robotic-assisted (RA) system for TKA, which can provide accurate bone resection and implant alignment based on preoperative planning and intraoperative guidance. This can potentially reduce the need for soft tissue release, improve implant alignment, and optimize knee kinematics [9]. Several robotic systems have been developed and used for TKA, such as Stryker’s Mako, Smith & Nephew’s Navio, Zimmer Biomet’s Rosa, and Curexo’s Cuvis Joint®. Among these, Cuvis Joint® is a fully automated robot that uses a cruciate-retaining (CR) implant design that preserves the posterior cruciate ligament (PCL), which may offer better proprioception and function than posterior-stabilized (PS) implants that sacrifice the PCL [10,11].

However, there is limited literature and evidence on the outcomes of CR robotic-assisted TKA using SA in obese and morbidly obese patients. Therefore, the aim of this study is to evaluate the clinical and radiological results of CR RA-TKA using SA in this population. We hypothesized that this technique would provide satisfactory outcomes with low complication rates.

## Materials and methods

This study, conducted at the Department of Robotic Joint Replacement, Sarvodaya Hospital, between June 2023 and November 2023, included 114 obese patients (215 knees) with pre-operative standing AP and lateral radiographs. The study also utilized CT scans of the involved lower limbs. Participants included patients with a BMI of ≥30 kg/m² (n=101) and morbidly obese patients with a BMI ≥40 kg/m² (n=13), all of whom had primary OA of the knee and underwent CR RA-TKA using the Maxx Meril CR knee (USA) with SA.

Ethical consideration

The study was approved by the Institutional Ethics Committee of Sarvodaya Hospital and Research Centre. It was conducted in accordance with the Declaration of Helsinki regarding research involving humans and its subsequent revisions, good clinical practice (GCP) guidelines, and other applicable national regulatory guidelines. The written informed consent form was taken from all the participants included in the study.

Pre-operative planning

Pre-operative planning for the procedure involved utilizing the Cuvis Joint® robotic system and the J-planner on the robot's console. The process included uploading the patient's CT scan onto the Robotic J-planner and registering the bony landmarks of the femur and tibia. This allowed for selecting the appropriate implant size and orientation to correct deformities and prevent anterior-posterior (AP) and mediolateral (ML) mismatch of the femoral condyle. The optimal implant size and position were determined by avoiding sagittal plane anterior notching and coronal plane ML overhanging.

Surgical technique

**Figure 1:**
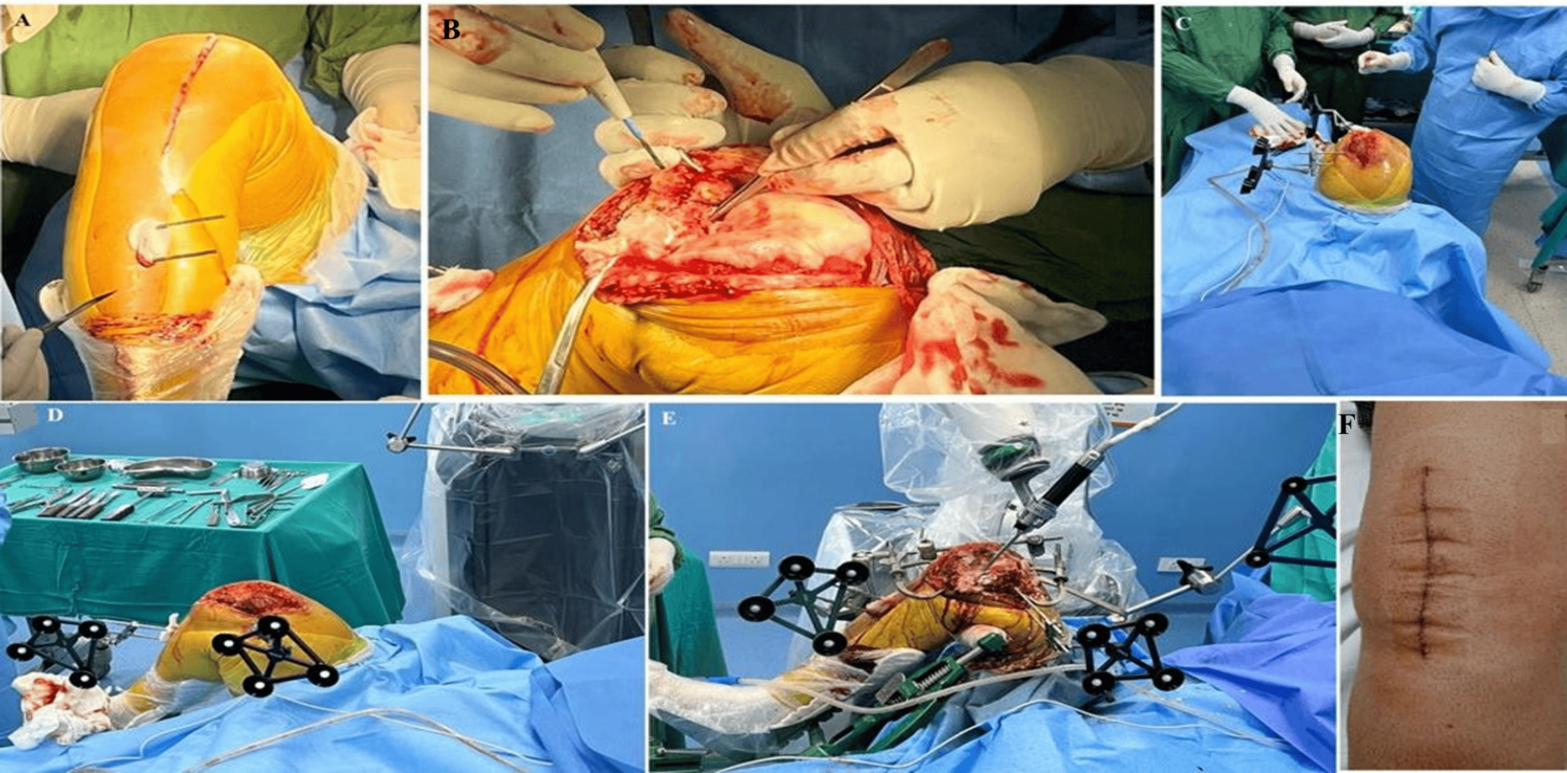
The steps involved in the surgical procedure. (A) Skin incision with two pins in the sagittal plane for robotic tibial array; (B) knee exposure in SA with patella subluxated lateral; (C) two femoral pins with a robotic array in coronal plane; (D) robotic tibial and femoral array mounted over the pin with intact quadriceps muscle with the knee in flexion; (E) femoral and tibial array, clamps, femoral distractor in situ along with robotic arm with burr; (F) skin closure in layers without any external suture.

The procedure was performed under spinal anaesthesia and femoral block without a tourniquet with SA. A skin incision was made slightly medial to the midline of the knee, and it was extended 4-5 cm above the superior pole of the patella to the inferior aspect of the tibial tuberosity. An incision was carried through subcutaneous tissue up to the deep fascia, followed by finger-assisted blunt dissection, and the remaining deep fascia and arthrotomy were completed with cautery (Figure 1A). The patella was subluxated laterally and held with a Hohmann retractor (Figure 1B). Tibial and femoral arrays were attached using two unicortical 3.5 mm tapered pins approximately 5 to 8 cm from the knee joint line, and visibility was confirmed throughout ROM (Figure 1C). The robotic tibial and femoral arrays were then mounted over the pins with the intact quadriceps muscle, with the knee in flexion (Figure 1D). The femoral and tibial arrays, clamps, and femoral distractor were positioned in situ, along with the robotic arm equipped with a burr (Figure 1E). Finally, the skin was closed in layers without any external sutures (Figure 1F).

Approved preoperative planning data were transferred to the robotic console, followed by registration of the femur and tibia, each with 40 surface mapping points aiming to achieve a root mean square error (RMSE) of <1. Achieving an RMSE of <1 is critical for the procedure’s success as it ensures high precision in aligning the implant components, minimizing discrepancies, and optimizing the overall accuracy of the surgery. The principle we follow is that wherever the cartilage is present, the probe has to be pierced slightly deeper, and wherever the underlying bare bone is there, we just touch the probe without embedding the probe deeper. At this stage, before bony marking, the femoral and tibial osteophytes are not removed. Registration points are marked and confirmed intra-operatively by circumnavigating around the osteophytes carefully, thus avoiding the large osteophytes so that the probe is kept on normal bone and not on overhanging osteophytes. Osteophytes are removed after bony registration because initial CT-based planning is done on the native knee along with osteophytes, so intra-operative initial femur and tibia registration through probe subsequently needs to be done in the same native anatomy, or else the registration points will change, leading to errors in marking, thus causing errors in resection and further errors in gap balancing and final implant positioning. After removing the accessible osteophytes, we balance the gap in a full range of flexion and extension.

**Figure 2:**
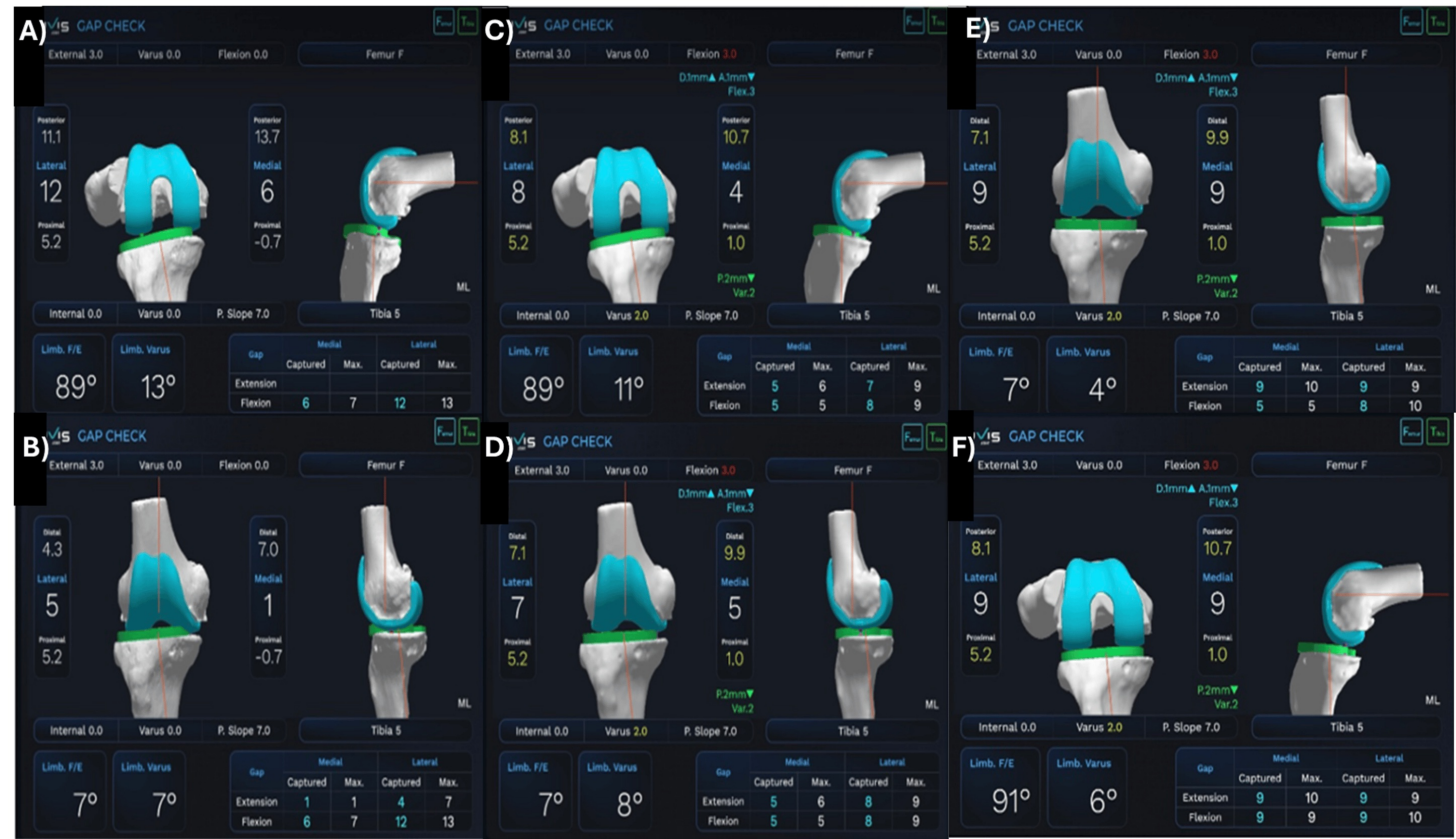
Robotic gap balancing workflow. Initial gap check (A) in flexion (B) in extension showing femoral implant in three external rotations (ER), no varus or flexion and tibial implant in 7° posterior slope (PS) with no internal rotation done or varus; preresection lateral gap balancing (C), (D) by adjusting femoral implant with 3° flexion, 1 mm more distal cut, 1 mm anterior down; tibial implant adjustment with 2° varus and 2 mm more proximal tibia cut; final gap check with the trial components implanted after bony resection and initial soft tissue balancing showing equal medial and lateral gap of 9mm in extension (E) and flexion (F) with 4° varus limb alignment.

Pre-resection (robotic burring) initial gap checks were done, and an assessment of coronal and sagittal plane deformity throughout ROM was performed. Necessary changes were made to the femur and tibial component positioning within defined limits to achieve intraoperative (Figure 2A-2F) gap balancing in 90° flexion to 180° extension. Either a natural tibial slope or 7° was kept while using the cruciate-retaining TKA implants. The gap was checked by keeping the lateral side as a reference (supposedly healthier and unaffected/less affected side) in varus knees since medial side tightness could be addressed later on after bony resection through adequate soft tissue releases and vice versa in valgus knees.

**Figure 3:**
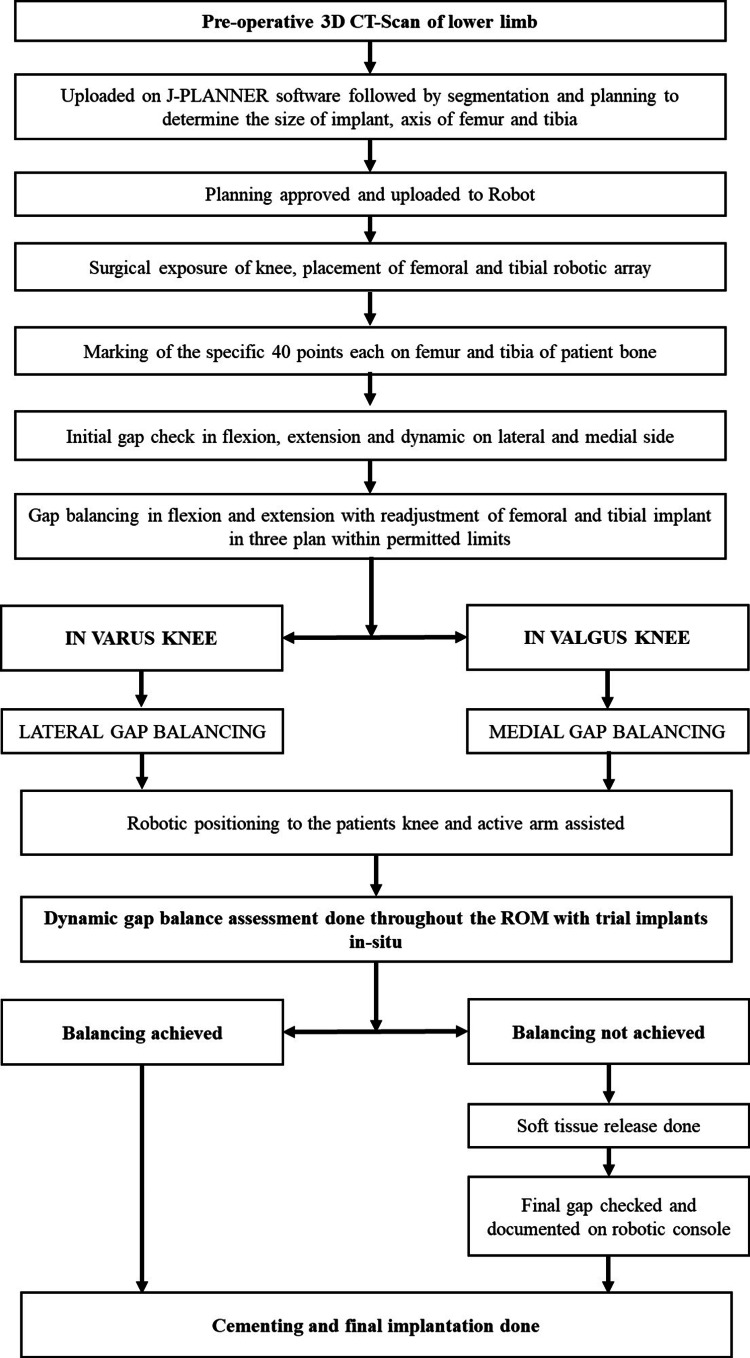
Flowchart of surgical technique.

Once the gap checking was done, the robot was attached using a femoral clamp, and then the robotic arm proceeded with bony resection as planned. In the present technique, full surface resection was opted for. The cut sequence can be changed or stopped during the burring process. After this, residual bone and osteophytes were removed. Post-resection (robotic burring) final gaps were assessed with trial implants in situ throughout ROM, and confirmation of symmetrical lateral and medial gaps in varying degrees of flexion as well as extension (zero-degree flexion to almost 130° of flexion) was achieved. Hip-knee-ankle (HKA) angle within 5° varus and 5° flexion was accepted based on the patient's pre-operative evaluation. We have accepted the mediolateral gap difference of +2 and −1 mm from the insert size. Once the knee was balanced, the femoral and tibial arrays were taken out, followed by patelloplasty with circumpatellar denervation. A 9-mm poly was used in each case. A pulsatile lavage wash was given, surfaces were dried, and components were implanted with cement. The closure was done in layers without draining using the staple-less technique. Patients were mobilized two to three hours post-surgery with walker support and started on ROM and strengthening exercises. Patients were discharged home on the post of day 3 or 4. All patients were evaluated with visual analogue scale (VAS) scores pre- and post-operatively on days 0, 1, 2, and 3 and using the AKSS clinical and functional scores at three- and six-month follow-ups (Figure 3).

## Results

**Figure 4:**
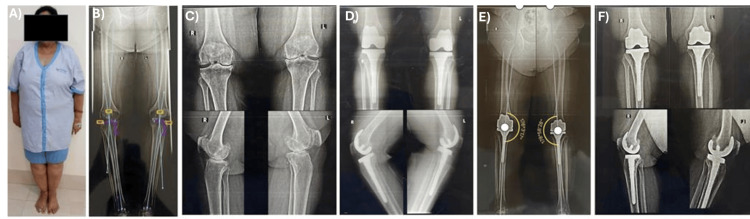
The post-operative orthoscanogram. (A) Morbidly obese patient undergoing CR RA-TKA with BMI 45; (B) pre-operative orthoscanogram with 12° varus deformity in both knees; (C) pre-operative radiographs - standing anteroposterior and lateral view; and (D) immediate post-operative standard anteroposterior and lateral view; (E) post-operative orthoscanogram showing hip-knee-ankle angle (right-4° varus and left-2° varus); and (F) post-operative follow-up radiograph at six months.

Out of 114 patients, 85 (75%) were females, and 29 (25%) were males, with an average age of 61.8 years (range 39-81 years). One case involved a morbidly obese patient with a knee deformity and a right flat foot. The patient's pre-operative orthoscanogram shows a 12° varus deformity in both knees. Pre-operative radiographs include standing anteroposterior and lateral views. Instant postoperative images display standard anteroposterior and lateral views. The post-operative orthoscanogram reveals the hip-knee-ankle angle, with a right varus of 4° and a left varus of 2°. A follow-up radiograph at six months is also included (Figure 4A-4F).

The demography and baseline characteristics of patients are presented in Table 1.

**Table 1:** Demographic and baseline characteristics of patients.

Demographic characteristics	n (%)
Total number of patients	114
Gender, n (%)	
Female	85 (75%)
Male	29 (25%)
Age, years, n (range)	61.8 (39–81)
Total knees	215
Bilateral knees	202
Unilateral knees	13
Average height, cm, n (range)	154.1 (139–177)
Average weight, kg, n (range)	83.1 (56.8–136)
Average body mass index (BMI), kg/m^2^, n (range)	34.8 (30–45)
Obese patients	101 (88.6%)
Morbidly obese patients	13 (11.4%)
Average coronal plane deformity knee, (range)	
Right	12.4° (2° varus to 30° varus)
Left	11.5° (8° valgus to 25° varus)
Average sagittal plane deformity knee, (range: hyperextended to flexion)	
Right	2.8° (−5° to 20°)
Left	3.2° (−5° to 20°)

We found satisfactory knee exposure using the SA technique, with no instances of having to terminate the procedure prematurely due to inadequate access. However, in morbidly obese patients with large thighs and deformities, it can be managed with some difficulty, and tourniquets are never used. There was no difficulty with patellar tracking, no cases of wound complications, wound infections, deep vein thrombosis, or pin track-related complications.

**Figure 5:**
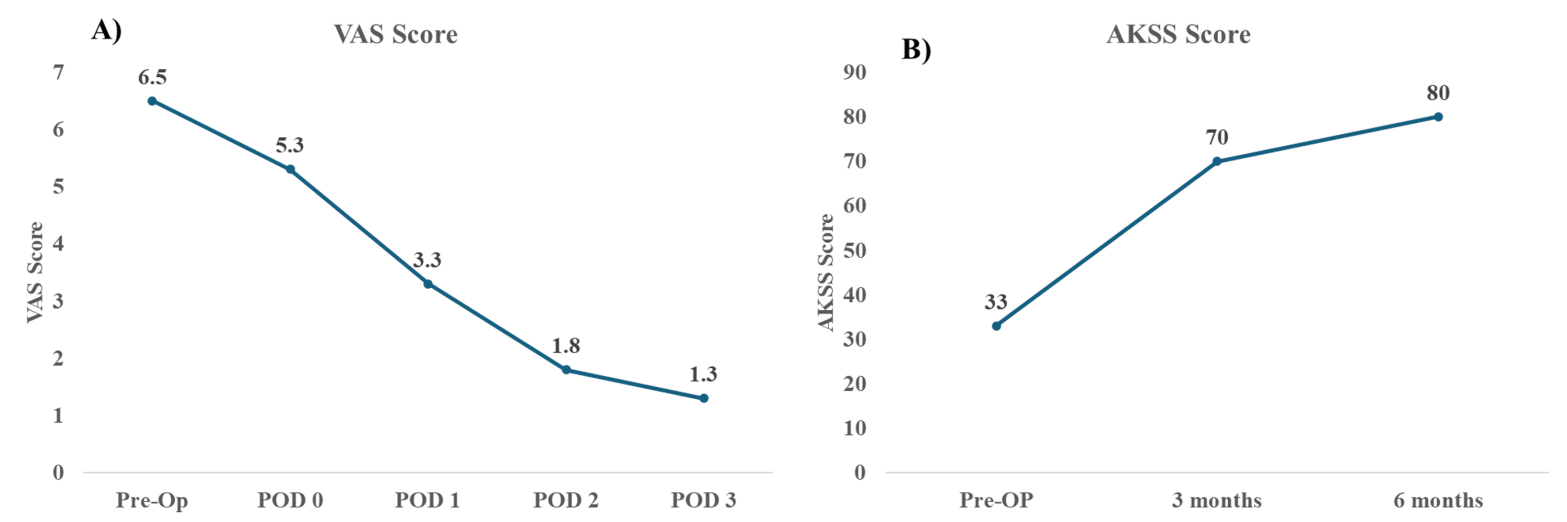
(A) VAS scores and (B) AKSS score.

Intra-operatively, two obese osteoporotic patients had medial collateral ligament avulsion fractures from femoral attachment while balancing with trial implants treated with cancellous screw fixation. Post-operatively protected weight bearing with a knee brace was advised. Another patient had a partial patellar tendon avulsion due to a patella issue, managed with a nonabsorbable suture. The VAS scores from pre-op and post-op days 0 through 3 were 6.54, 5.3, 3.32, 1.87, and 1.3, respectively. (Figure 5) The AKSS showed improvements from a pre-operative average of 33.9 (15-55) to 70.7 (52-84) at three months and 80.9 (68-92) at six months.

Four patients, two unilateral and two bilateral TKA cases, underwent tibial stem implantation. Out of four, two patients had severe osteoporosis with a T-score ≤ −3.5 and severe coronal plane deformity of more than 20; the other two patients were morbidly obese with a BMI of 40.1 kg/m^2^.

The quadriceps recovery was faster in all patients. Ninety percent of the patients (102 patients) were able to do an active straight leg raising test on day 1, and 96% of patients (109 patients) were on day 3. By day 3, 92 patients (81%) were able to walk without support, and 28 patients (25%) were able to climb stairs.

Only 10% of patients (11 patients) showed 10° quadriceps lag after day 3, and this was evident in knees with more than 10° flexion deformity and pre-operatively weak quadriceps.

At six-month follow-up, 80% of knees (172 knees) exhibited an exceptional ROM with a flexion arc ≥ 120°, and 20% of knees (43 knees) had a ROM between 90° and 120°. The mechanical alignment of the lower limb was adequate, with an average varus angle of 3.8° (range: 3° to 5°). The component placement and cementing appeared satisfactory at the six-month follow-up radiograph in all patients.

## Discussion

The subvastus approach in TKA is a muscle-sparing technique that aims to preserve the quadriceps muscle, leading to faster recovery and reduced post-operative pain compared to other approaches.

However, obese and morbidly obese patients may be considered unsuitable for this approach due to potential challenges in exposing the surgical site, which can increase the risk of complications such as medial collateral ligament sprain or patellar tendon avulsion [12]. This study found that separating the vastus medialis from the medial intermuscular septum resulted in easy lateral subluxation of the patella. In a study by Mathison [13], it was reported that morbidly obese patients faced more difficulties during patelloplasty compared to obese patients. We also found that morbidly obese patients had more difficulty undergoing patelloplasty than obese patients, so we performed the same after completion of bony cut and osteophytes removal, which made patella manipulation a little easier.

Young et al. [14] considered a large thigh girth (>55 cm) as a contraindication to the mini-subvastus approach because of the difficulty in eversion of the patella. We believed that the exposure would not be difficult as the anatomy of the quadriceps in the obese and the non-obese patients is the same, and the eversion of the patella, although difficult initially during the approach, would not be so difficult after the osteotomy of the femur, as this would have effectively relaxed the quadriceps muscle.

Component alignment is a major concern in minimally invasive TKA, particularly in obese patients [15]. However, in the current study, no component misalignment was observed, which can be attributed to the utilization of robotic assistance to aid in the intraoperative execution of planned bony cuts and achieve accurate positioning of the component. We believe that component malalignment can be avoided by careful identification of the anatomical landmarks and the use of standard surgical jigs.

The subvastus approach in the present study did not violate the extensor mechanism, providing intrinsic stability to the patellofemoral joint. Patelloplasty with circumpatellar denervation resulted in satisfactory post-operative patellofemoral tracking and reduced pain, facilitating rehabilitation and reducing the requirement for analgesics [16]. In a similar study using the subvastus approach in obese patients by Shah et al. [6], the authors noted that they did not have to evert the patella primarily at all but only displaced it laterally and that it can be displaced laterally easily if the vastus medialis is released adequately from the intermuscular septum. The synovial division also helped in the lateral displacement of the patella. It was possible to flex the knee more (if the knee was stiff earlier) by the release of the vastus medialis from the septum and the removal of all osteophytes. The knee was "debulked" by removing the osteophytes and releasing the soft tissue, making the exposure easier. After the femur and tibial bone cuts are performed, patellar eversion for resurfacing or debulking (the removal of protruding superior and lateral osteophytes) can be accomplished rather simply since the quadriceps become more relaxed. Repositioning the knee in different degrees of flexion and extension is necessary while performing the surgery through a tiny incision. Although all of the knee's components may not be seen at the same time, sufficient exposure is achieved to complete every step of the procedure. The fundamental components of the subvastus technique are the symbiotic usage of retractors and the employment of a moveable skin window to complete the operation's successive steps.

The subvastus technique is found to be less invasive than the standard approach, as it maintains blood supply via the intramuscular descending genicular artery, reducing the risk of vascular complications [17]. Skin necrosis can be avoided by careful subfascial dissection and by not undermining the lateral flap. As is well known, the vascularity of the knee is lateral-based, and by keeping the lateral flap thick, skin necrosis can be avoided.

In obese and morbidly obese patients, the incidence of complications related to TKA is notably high, even with the parapatellar technique. These complications can include wound complications, avulsion of the medial collateral ligament, avulsion of the patellar tendon, deep joint infection, and deep vein thrombosis [18-19]. The present study did not observe any skin-related complications or thromboembolic events in obese and morbidly obese patients, which may be attributed to the careful dissection of the medial soft tissue skin flap with subcutaneous deep fascia release during the procedure. Favourable outcomes were also aided by early patient mobilization, and TKA was performed without the use of a tourniquet. As per Hakim et al. [20], one patient had skin necrosis in the morbidly obese, one patient in the obese, and three patients in the morbidly obese group had thromboembolic events. Two patients in the obese group and four patients in the morbidly obese group suffered from superficial wound infection in the immediate postoperative period.

Recently, Schroer et al. [21] published their results of 600 primary total knee arthroplasties performed through a subvastus approach. Follow-up was short-term, averaging 28 months. A historical group of 150 total knee arthroplasties performed through a standard medial parapatellar arthrotomy was used as a control. The rate of major complications in the subvastus group was found to be associated with surgical experience, as the rate was reduced by 16% for each additional 50 procedures performed. Mean knee flexion at one year averaged 125° in the subvastus group and 114° in the traditional group. The average operative time was initially higher in the subvastus group but decreased with experience, so it was less than that of the traditional group in the last 400 subvastus procedures performed.

Revision surgeries are frequent in obese patients due to early aseptic loosening and periprosthetic fracture. However, in the present study, no cases of aseptic loosening or adverse events were observed during the follow-up period [22].

The use of robotic-assisted TKA has advantages and disadvantages. While it can result in marginally longer surgical time, it can also aid in the accurate positioning of components and reduce complications associated with the subvastus approach [23]. One of our patients fell on the fourth postoperative day, resulting in an MCL sprain on the right knee and a patellar tendon tear on the left side. The patellar tendon was reconstructed using a peroneus longus tendon graft. The right knee's MCL was treated conservatively. The patient was immobilized with a knee brace for six weeks, followed by gradual rehabilitation. At six months, both knees had a range of motion of 0-90°.

In this study, no robotic array pin issues were seen during or after surgery. Pins were placed in the metaphysis for the femur and smaller pins in the diaphysis for the tibia. A significant concern in robotic TKA is femoral/tibial fractures because of the stress caused by the pinhole stress. Yun et al. [24] and Baek et al. [25] recommended periarticular pin placement because the bone at this site is more robust to torsional and bending stresses than the diaphysis. Kazmers et al. [26] advised the smaller pins to prevent this complication. Pin-site infection is another specific complication of the tracker pin that may require antibiotics and dressing for an additional duration. However, the incidence of pin site infection was reported to be low in general (0.47%).

Using SA for CR RA-TKA led to lower intraoperative blood loss (around 200 ml, ranging from 150 ml to 400 ml) in obese and morbidly obese patients, a more favourable outcome compared to other studies.

Surgical techniques have limitations that result in specific restrictions on their use, like in patients who are shorter than 140 cm, have severe osteoporosis with a T score of −4, or have significant deformities exceeding 20° in the sagittal plane or 20° in the coronal plane. It is worth noting that this study is non-randomized. However, with over 100 patients, it provides a strong foundation for a potential large-scale, long-term study comparing outcomes between obese and non-obese patients in the context of a pioneering approach to cruciately retaining robotic-assisted TKA using a subvastus approach.

## Conclusions

The short-term follow-up study demonstrates the effectiveness of RA CR-TKA using the SA in obese and morbidly obese patients. This approach provides adequate intraoperative knee exposure even in obese and morbidly obese patients with minimal blood loss and less pain in the immediate postoperative period, thus enabling early mobilization. Additionally, it improves knee range of motion and patellar tracking, leading to a faster recovery and effective rehabilitation. Importantly, despite minimally invasive procedures, the robotic arm assistant maintains proper implant positioning and limb alignment. These findings suggest the potential feasibility of CR RA-TKA in morbidly obese and obese patients, utilizing the subvastus approach without a tourniquet. As with any procedure, there is a definite learning curve associated with the subvastus approach. The senior author’s technique and indications have evolved with time and experience. While these short-term results are promising, longer-term studies are needed to confirm these findings and fully understand the durability and long-term outcomes of this approach.
